# Supplementary material for: Saccharomyces boulardii (CNCM I-745) improves intestinal damage in sepsis by remodeling intestinal flora structure
Source: Front Cell Infect Microbiol. 2025 Jul 31;15:1602792. doi: 10.3389/fcimb.2025.1602792 (PMC12350329; doi:10.3389/fcimb.2025.1602792)
Supplement: Supplementary file 1 [file DataSheet1.zip › summary/summary/8_Advanced_analyse/2_PICRUSt2_result/KEGG/Group.DW0_vs_CW0_vs_BW0_vs_AW0/PICRUSt2_KEGG_level3_Group.DW0_vs_CW0_vs_BW0_vs_AW0_diff.pdf]

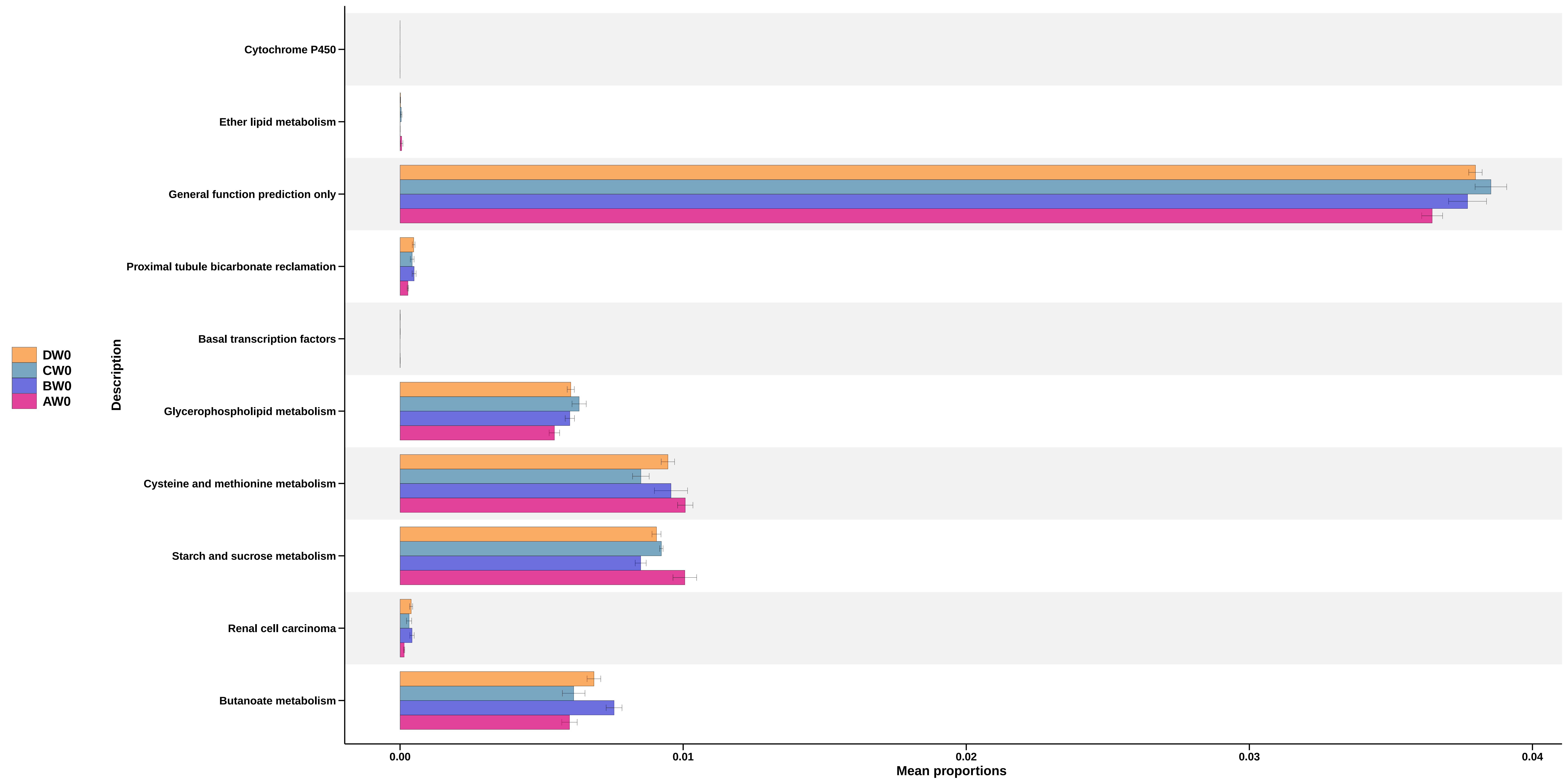

Cytochrome P450
Ether lipid metabolism
General function prediction only
Proximal tubule bicarbonate reclamation
Basal transcription factors
Glycerophospholipid metabolism
Cysteine and methionine metabolism
Starch and sucrose metabolism
Renal cell carcinoma
Butanoate metabolism
DW0
CW0
BW0
AW0
Description
0.00
0.01
0.02
0.03
0.04
Mean proportions
